# Multiple qualitative procedures to elicit reverse culture shock experience^☆^

**DOI:** 10.1016/j.mex.2019.12.007

**Published:** 2019-12-16

**Authors:** Walanchalee Wattanacharoensil, Suwadee Talawanich, Laddawan Jianvittayakit

**Affiliations:** Tourism and Hospitality Management Division, Mahidol University International College, Thailand

**Keywords:** Multiple qualitative procedures to elicit RCS experience, W-curve study, Memory recall, Qualitative methods, Educational tourists, Working tourists, Thai youth

## Abstract

This study elaborates the research design and methodology to investigate the reverse culture shock (RCS) experience of young Thai tourists in the tourism field. Taking the worldview position of relative ontology and interpretivist/constructivist paradigms, this paper employs qualitative design using multiple research methods, namely, essay writing, graph plotting and semi-structured interview. Essay writing and graph plotting are initially used as pre-interview activities as part of the memory recall procedures. This stage is important because it helps curtail memory distortion and enrich insight into the participant’s past RCS experience. The semi-structured interview with young Thai tourists is subsequently conducted to elicit individual perceptual and emotional experiences and coping mechanism after they returned home. The three research methods complement one another to draw out rich travellers’ experience in the tourism study and can be beneficial to the extended disciplines of social science and psychology.

Advantages of this article include:

• Practical and feasible processes for a qualitative study in social science and psychology, particularly when recall of memory is involved.
• The ability to gain enriched information of experience.
• The ability to elicit emotional aspects from the study of experience through graph plotting.

## Graphical abstract

**Figure:**
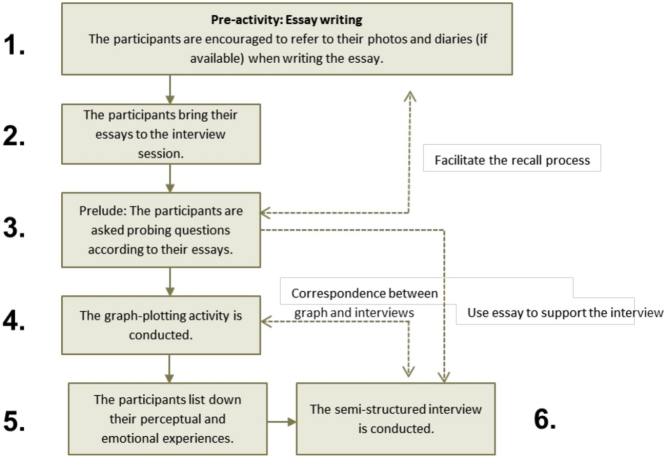

**Specification Table**

**Table:** 

Subject Area:	Social Sciences
More specific subject area:	Reverse culture shock in tourism
Method name:	Multiple qualitative procedures to elicit RCS experience
Name and reference of original method:	Adapted from Ellis, J., Amjad, A., & Deng, J. (2011). Interviewing participants about past events: The Helpful Role of Pre-Interview Activities. *In Education, 17*(2), 61-73. Ellis, J., Hetherington, R., Lovell, M., McConaghy, J., & Viczko, M. (2013). Draw me a picture, tell me a story: Evoking memory and supporting analysis through pre-interview drawing activities. *Alberta Journal of Educational Research, 58*(4), 488-508. Gullahorn, J. T., & Gullahorn, J. E. (1963). An extension of the U-Curve hypothesis. *Journal of Social Issues, 19*(3), 33–47. Gaw, K. F. (2000). Reverse culture shock in students returning from overseas. *International Journal of Intercultural Relations, 24*, 83–104.
Resource availability:	Not applicable

## Background of the study

A growing number of Thai youths assuming the role of tourists through exchange student and working holiday programmes have recently been investigated. However, youth tourists claim to face reverse culture shock (RCS) during their return to their home country (the re-entry phase), thereby negatively affecting the readjustment process to the home culture (see, for example [1,2,[3], [4], [5], [6]]).

Although the RCS phenomenon is widely proven in numerous countries, the insights into the RCS amongst youth tourists who participate in exchange student (educational) and working holiday programmes have been rare and remain underexplored. Most diverse RCS studies in youth focus on full-time students (see, for example, [3,5,7,8]) and short-term expeditionary programmes (see [1,2]). To address this limited knowledge, this study firstly aims to investigate RCS among Thai youth tourists by exploring the perceptual and emotional stages and the coping strategies that occur after their return to their home countries.

**Fig. 1:**
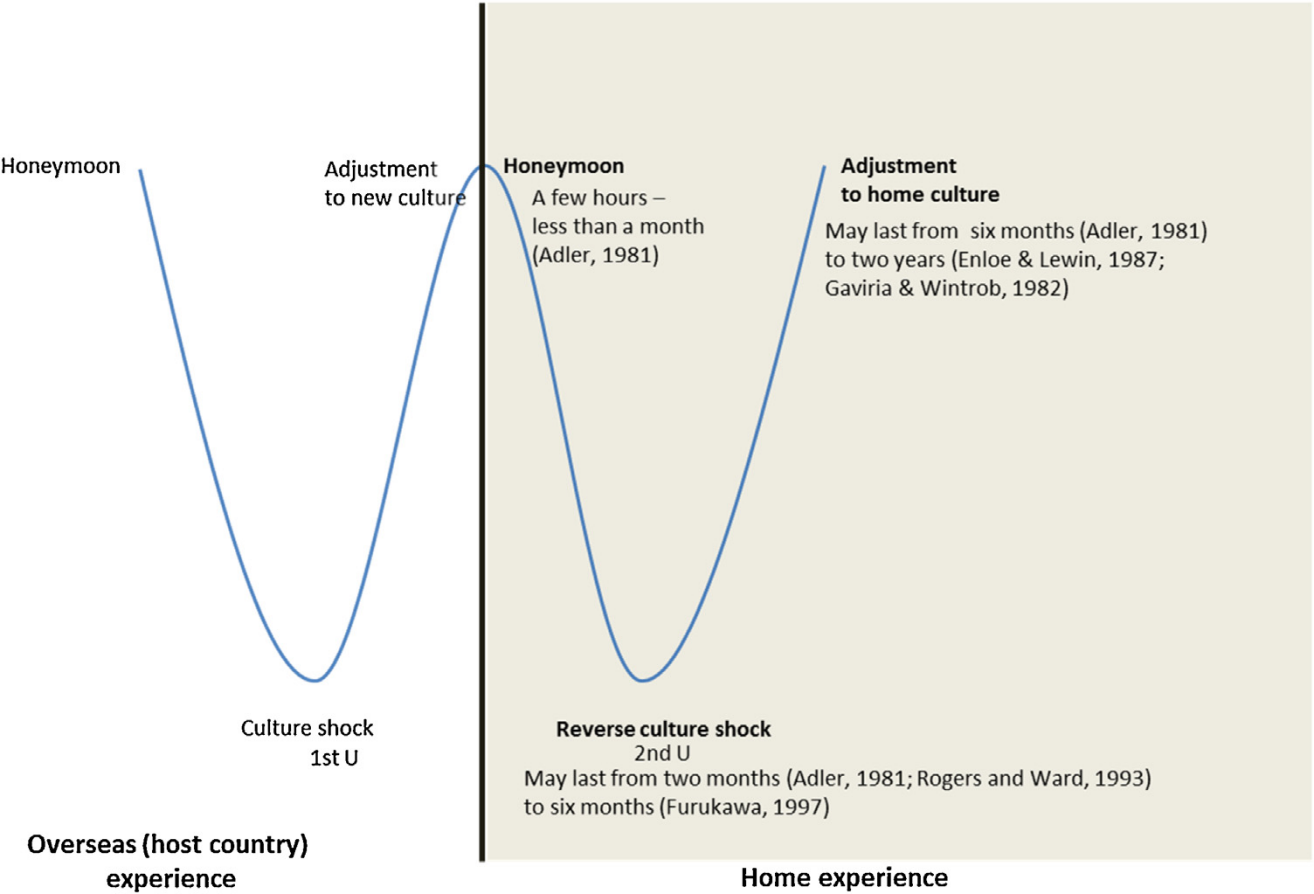
W-curve proposition adapted from Gullahorn and Gullahorn [4].

The present study selected the W-curve proposition [4] as framework for this study owing to its explanatory power on the relationship between ‘timeframe’ and the ‘emotional stage’. Gullahorn and Gullahorn [4] introduced the W-curve proposition (extended U-curve adjustment theory of culture shock by Lysgaard [9]) to elucidate the RCS phenomenon among American youths/students. The extended U-curve demonstrates that the returnees are likely to encounter the second U-curve in their home country [4]. The second U-curve comprises (1) honeymoon, which represents good initial adjustment; (2) RCS, which refers to an adjustment ‘crisis’ and (3) adjustment to *home* culture [2,9]. Fig. 1 illustrates the W-curve proposition.

Previous RCS studies are conducted in Western and Asian contexts and verify the explanatory power of W-curve proposition in diverse degrees. The studies conducted in the UK, Germany and Turkey support the full or partial existence of the second U-curve (see [1,2,6]). By contrast, RCS partially existed amongst Taiwanese and Sri-Lankan youths [7]. Given the context-based applicability of W-curve proposition, our paper aims to investigate the RCS phenomenon by applying the W-curve proposition to the Thai context and examining the extent that the traditional W-curve proposition can explain the re-entry phase and the RCS symptom among Thai youths.

Hence, pre-interview activities combined with a semi-structured interview amongst the 26 participants are employed for data collection and analysis.

## Research design

This paper aims to investigate RCS experiences amongst Thai youth tourists who travelled overseas from two main categories, namely, educational and working tourists. Given that this study explores the RCS experience of individuals, the researchers posit that experience is not characterised by a single reality but comes from multiple perceptions and experiences of different individuals. Thus, the authors are guided by the theoretical view of relative ontology (relativism), whereby each person perceives the world through his or her own lens and perception. The reality under relativism can be represented by shared meanings and the ways that each individual manifests and perceives his or her experiences [10]. In this study, the experiences of RCS among youth tourists are based upon culturally-influenced values, assumptions and beliefs [11] that form and influence their points of view and perceptual and emotional experiences.

Interpretivism/constructivism is selected as the research paradigm to respond to the chosen ontological standpoint. Social constructivism posits that individuals seek understanding of the world and develop subjective meanings of their experiences toward certain objects or things [12]. Following this epistemological standpoint, the researchers look into the complexity of views and multiple meanings of RCS experiences rather than a merely narrow and surface viewpoint [12]. Therefore, the qualitative research design is chosen to complement the selected worldview, given that this approach allows the researcher to acquire rich meaning and more dimensional explanations from respondents. Details about the design and the selected research methods are further explained in the following section.

**Fig. 2:**
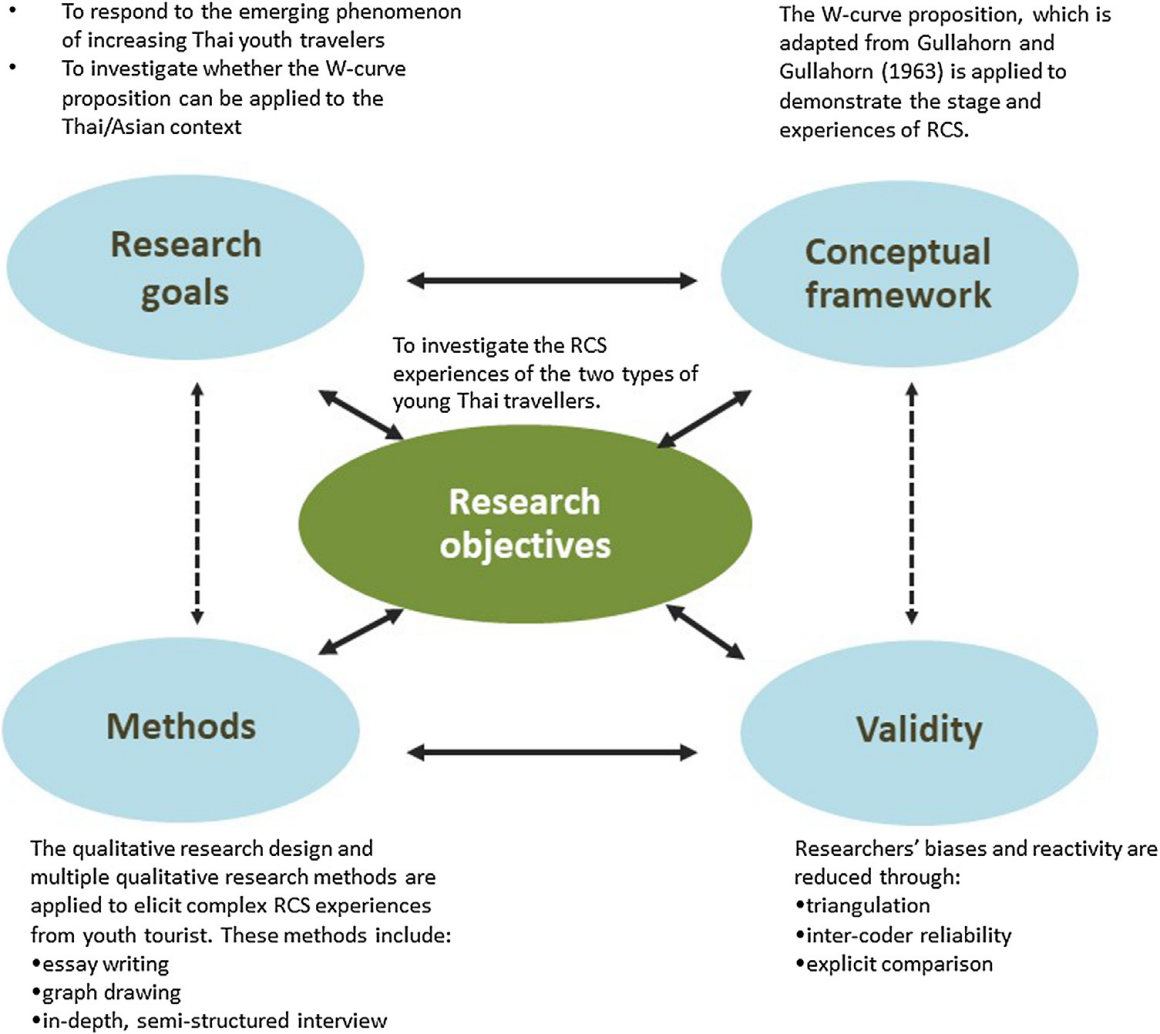
An interactive model of research design (adapted from Maxwell [13]).

In particular, the researched apply *an interactive model of research design* by Maxwell [13] that comprises five interrelated elements to demonstrate and ensure research coherence. Fig. 2 illustrates the application of the interactive model of research design.

### Research goals

Maxwell [13] mentions that the research goal provides the reason why the study is worthwhile. This study *responds to the remarkably emerging phenomenon* in Thailand where a number of Thai youth tourists who go overseas to work, study and travel have dramatically increased. However, the RCS experience can lead to negative psychological effect when they return home. Therefore, enriching insights into this topic improves understanding about the youths’ psychological stages and their coping mechanism upon return. In addition, this study theoretically contributes to the application of W-curve proposition because it extends the field of investigation into the Asian context.

### Research questions/objectives

Research questions/objectives indicate the particular outcome that researchers want to learn or understand [13]. The researchers of this study generally aim to investigate the RCS experiences of the two types of Thai youth tourists. Accordingly, the researchers raised the three specific objectives, namely:

• to explore the perceptual and emotional stages that Thai returnees experience after completing their work and holiday and educational experience overseas;
• to investigate how the two groups of Thai youth tourists cope with the perceptual and emotional stages; and
• to identify the degree to which the RCS frameworks proposed under western context can explain the phenomenon in Asian context, with a focus on Thai youth travellers.

### Conceptual framework

The W-curve proposition is employed in this study. Gullahorn and Gullahorn [4] proposed the W-curve to explain the occurrence of RCS. W-curve proposition represents the overall stages that the youth returners experience from their arrival in a foreign country to their return to their home country (see Fig. 1). Therefore, this framework is deemed suitable for the RCS study in Thailand.

### Methods

This research applies the qualitative research design involving multiple qualitative research methods that allow researchers to capture rich and complex views of experiences from respondents. These multiple methods include essay writing, graph drawing and in-depth/semi-structured interview.

### Validity

Validity indicates that researchers may provide false claims when conducting research. This research feature involves two broad types of threats, namely, researcher bias and researcher reactivity (the effect of the researcher on the setting or toward the respondents) [13]. To minimise threats on validity, the researchers often discuss and challenge each other’s thoughts and ideas throughout the research processes to minimise researchers’ biases. Moreover, triangulation and intercoder reliability are applied during the data collection and analysis. Explicit comparisons with previous studies on the same topic are also carried out.

## Research methodology and methods

The research methods of this study incorporate the pre-interview activity concept proposed by Ellis et al. [14] and Ellis et al. [15], along with the methods obtained from the previous RCS studies (see [1,2]). The researchers modified the tools to complement the research objectives. The three key research methods comprise essay writing, graph drawing and the semi-structured interview.

For this study, recalling memory through the use of visual items (i.e. drawings, diagrams, photographs and listings) is a crucial step that enables individuals to remember relevant experiences, particularly when one wants to review past events from a long period of time [14]. In addition, the discussion on the visual items can function as a complete checklist of necessary details to ensure that the participants would provide sufficient information [15]. The memory recall process would require a person to recall his or her RCS experience, as well as his or her perceptual and emotional stages upon returning home from overseas. The stage of memory recall is carefully designed to curtail or lessen memory bias and distortion during the investigation [16].

During the first stage of memory recall, three open and broad questions were asked to the participants to trigger their experiences, perception and emotion. They were asked to write down half-page to one-page essay about their experience in different timelines, namely, one month before and eight months after the returning phase to their home country (Thailand). Moreover, the participants were encouraged to refer to their own diaries and photos, which would help them reminisce their experiences before and after their return. The pre-activity essay would be used for further content analysis and as preliminary material for the interview. This essay writing technique was adopted from the activity of writing letter expressing re-entry experiences, which is conducted in previous RCS literature (see [1,2]).

**Fig. 3:**
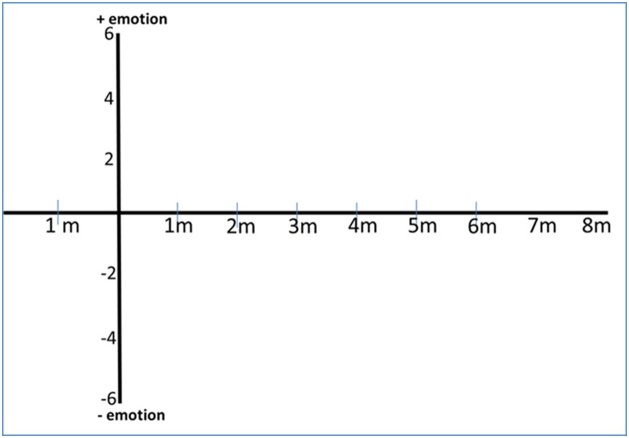
Graph plotting scale.

In the next step, the researchers conducted another recalling memory activity to facilitate the participants to look back on their memory lane before the interview. A participant would be invited to attend the pre-activity session and bring the essay along. The researchers conducted a brief discussion with regard to the essay and asked probing questions to refresh the participants’ memory about the RCS. This initial talk would take for 10 to15 minutes. Afterward, the participants were asked to draw a graph to represent their feelings after they return to their home country at different points in time, starting from one month prior to return until eight months upon return. The researchers applied Ellis et al. [15] and Ellis et al.’s [14] concept of a graph-drawing activity to facilitate the recall of perception and emotion regarding past events. The graph was drawn in relation to the two-axis relationships (timeline and positive/negative emotion). The graph-plotting activity is adopted to provide the visual evidence for investigating the degree of W-curve proposition matches the participants’ perceptual and emotional stages based on the western framework. During the graph plotting activity, each participant was asked to carefully reflect on their feelings at each stage. The participants could also bring their diary and photo album to the session to help them recall their perception and emotion. No interruption or disturbance occurred during this stage. The researchers merely responded to questions that arise during the graph-plotting activity (Fig. 3).

**Fig. 4:**
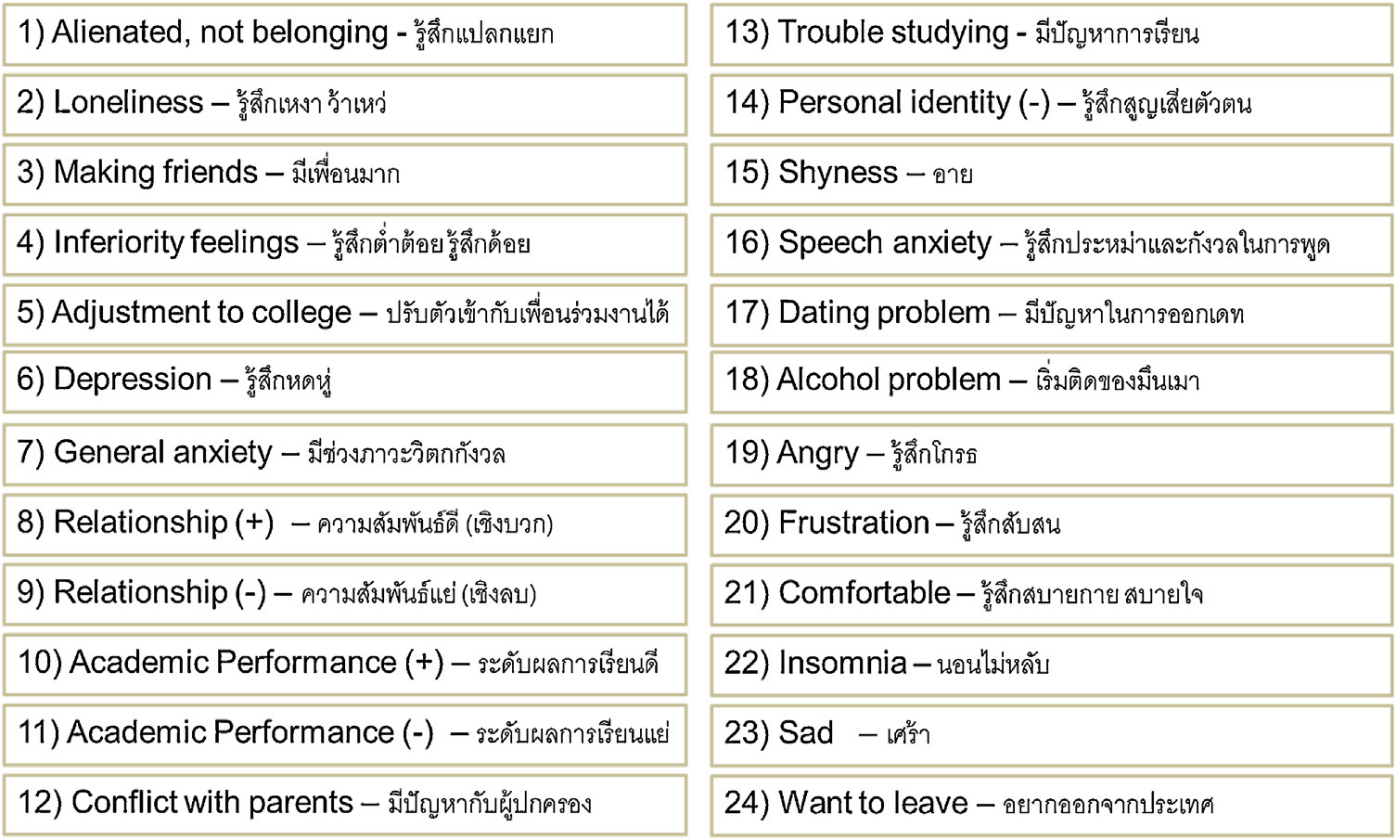
List of perceptions and emotions experienced at different stages (adapted from Gaw [3]).

After the participants drew the graph according to their emotional stage at each timeline, they were asked to plot down the related emotions and perceptions onto the graph by writing down the number that reflects his or her emotion at each stage. The list of emotions and perceptions has been taken from the questionnaire items used in the RCS study of Gaw [3]. Moreover, the participants were asked to recall other types of emotions or perceptions that they expressed but were not included in the list. Such item was added in number 25. Fig. 4 describes the details of emotions and perceptions.

The aforementioned procedures served as the pre-interview activities. The participants took approximately 45 minutes to one hour to draw the graph and plot their level of emotion and perception. Subsequently, the semi-structured interview was conducted. The interview aims to clarify and probe the details regarding the situations, perceptions and emotional stages, as well as their coping mechanism, during their return. The interview was conducted to supplement the graph that was previously drawn and thus allow the participants to elaborate their thoughts and feelings at each stage. Furthermore, the interview questions were customised to reveal the emotion and perception stages that the participants experienced and their coping strategies upon returning to their home country.

The graph plotting was designed based on the emotional and time scales, which were adopted from Gullahorn and Gullahorn [4], including perceptual and emotional elements that were adopted from Gaw [3]. Interview questions were created to pursue the three main research objectives. Moreover, the researchers adopted the previous RCS studies suggesting the existence of perceptual and emotional stages (see, for example, [1,3,7,17,18]), as well as the questions regarding their coping strategies [19,20] and the RCS framework of W-curve proposition [4].

The interview questions required further explanation for the graph, and the additional items were used to tackle the third research objective (the application of western RCS frameworks in the Asian context). One example of the probing questions asked in the interview is, ‘Explain why you drew the graph in the period of […]. Specifically, what did you feel during that stage?’ The probing questions covered various dimensions that were affected based on the participants’ perception during the adaptation stages upon their return. These dimensions included relationship, family, work and daily lifestyle. Appendix 1 presents several examples of the graph and the interview questions. Given that all interviewees and researchers are Thai natives, the memory recall procedures and tools (i.e. probing process during the interview, verbal explanation of the overall procedures and written instructions for writing an essay and plotting a graph, development of the interview questions) were written in Thai language to minimise the language barrier when one’s information and expression are provided. The interview was recorded and transcribed verbatim.

**Fig. 5:**
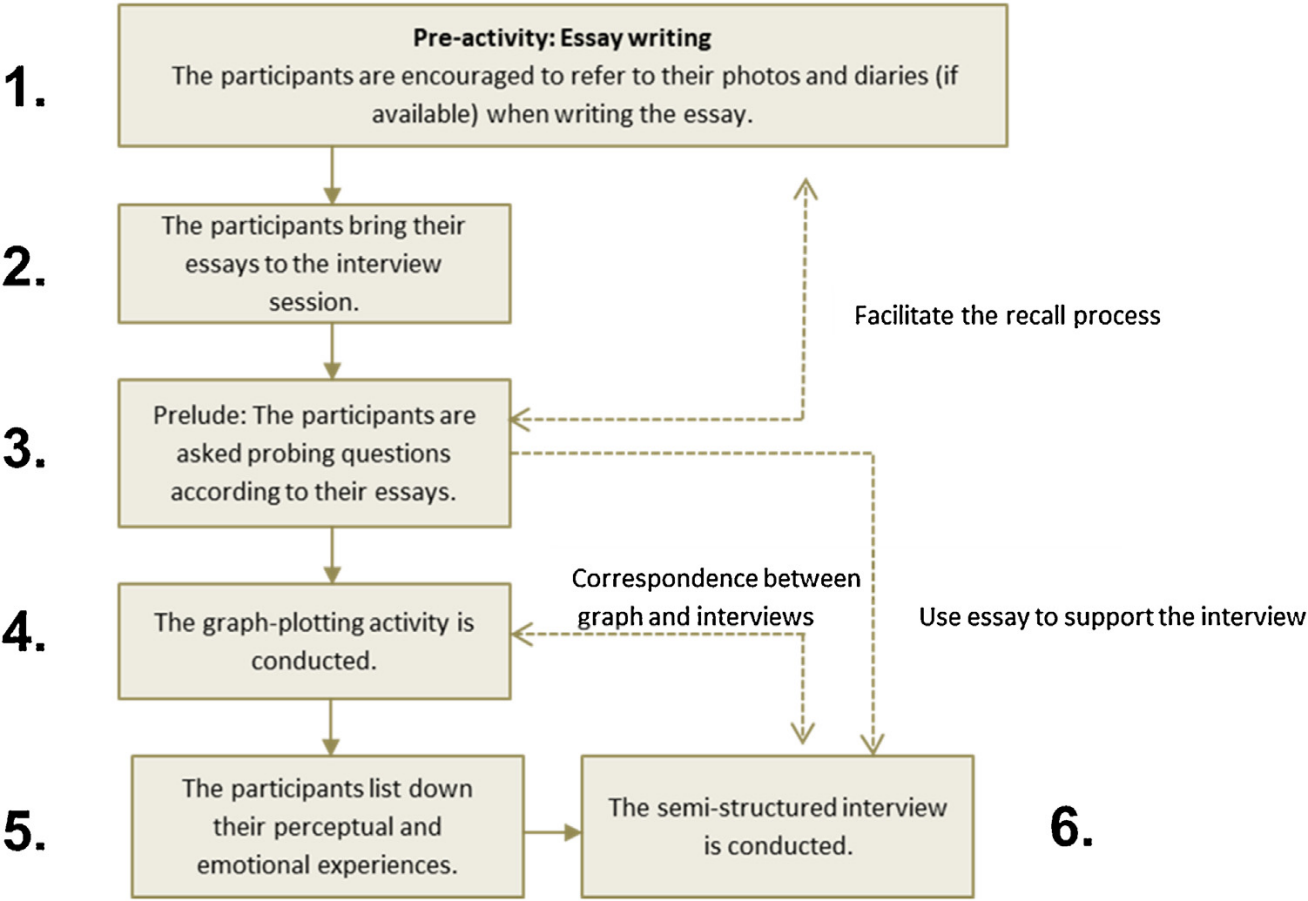
Process flow of research procedures.

In conclusion, six-step procedures were involved in the recall process, particularly for investigating RCS in the study. Overall, the entire process took approximately 90 to 120 minutes. Details about the six-step procedures and the flow of research procedures are described as follows: (Fig. 5)

*Pre-activities for the recall process* (approximately 30 minutes, at their own environment)

1 The pre-interview and story-telling through essay writing

*Pre-activities for the recall process* (approximately 45 minutes, with the researchers)

2 The participants take their written essay about their overseas experiences, together with their diaries and photos, to the interview session.
3 According to their written essay, the probing questions are asked to the participants.
4 The participants are asked to plot the graph to indicate the relationship between time and their emotional stage.
5 The participants are asked to list down the emotions and perception (perceptual and emotional list, adapted from Gaw [3]) that they expressed during the interview.

*The interview session* (approximately 45 minutes to one hour, with the researchers)

6 The semi-structured interview is carried out.

## Sample size and data analysis

The data were gathered from 26 Thai youth travellers who have returned from overseas programmes. A total of 12 males and 14 females aged 16 to 22 years old participated in the study. Fifteen youths travelled to the United States (US), two went to South America whilst the rest visited Western Europe. The two categories of youth tourists were selected based on their travel purpose, namely, working tourist and educational tourist. The latter category was further divided into two subgroups, namely, 1) those who attended a one-year exchange program (13 youths) and 2) those who short-term exchange program (6 youths). Thus, the number of working tourists equals 7 youths. To determine the participants who were eligible for the interview process, the three following pre-screening conditions were employed.

• They must have stayed overseas in a minimum of three months.
• They must have spent time in a non-Asian country.
• They must have returned to their home country (Thailand) for more than six months and up to five years.

The first two conditions aim to ensure that the participants have spent sufficient time and have a chance to experience culture shock in the country where the cultural context is dissimilar to his or her own (see further [16]). The last condition aims to enhance the probability that the RCS cycle is completed or continued after he or she returns.

The multiple stages of data collection (i.e. essay writing, drawing graph and transcribing the interview verbatim) serve as the source of data for the analysis process. Apart from the essay and graph that were written in Thai, the Thai interview content was also transcribed directly. All the data were then analysed in Thai language. Thus, the first-hand meaning of the Thai youth tourists’ experience and the sociocultural interpretation could be fully comprehended by the Thai-native researchers who exhibit good command of Thai and English languages. The researchers organise the data into themes and subthemes through the support by NVivo 11 software. Themes were indicated based on the pattern of data according to the perceptual and emotional aspects of respondents on RCS.

The coding process of each interviewee’s script was separately conducted by two researchers. Subsequently, the codes from the two researchers were crossed-checked to ensure inter-coding reliability, thereby obtaining agreement on the majority of codes. The researchers discussed about uncertain codes and decided a consensus until all the codes were agreed upon. Certain sentences that were quoted into the manuscript were translated to English by the researcher who previously coded such sentences. Next, another researcher verified the translation equivalence that refers to the lexical, idiomatic, and syntactical similarity gained across diverse languages [21] of the quoted sentences. The effort to address the possible equivalence concern allowed the researchers to obtain trustworthiness, particularly in the cross-cultural research [22]. Although the W-curve of RCS was used as a broad theoretical framework of the study, the researchers allowed for any new emerging concepts.

## Trustworthiness of the study

To ensure the trustworthiness of the study, the researchers followed Lincoln and Guba [23]’s categorisations and endeavoured to warrant the four aspects of trustworthiness including credibility, transferability, dependability and confirmability.

To ensure the study’s *credibility* that refers to the confidence in the ‘truth’ of the findings, the researchers employed method and data triangulation techniques to cross-check and validate the results. The three methods (i.e. essay writing, graph plotting and interviewing) that yield three data types were compared and contrasted to ensure the confidence level, minimise data contradiction and ensure data sufficiency prior to analysing their meaning. *Transferability* that refers to the ability to show that the findings have applicability in other context is done through logical and in-detail explanation of the research design and procedures. *Dependability* that reflects how the findings can be repeated is also achieved through the detail explanation of research procedures, along with documentations of the findings and researchers’ thoughts and ideas. Lastly, *confirmability* represents the degree of neutrality or the extent to which the findings of a study are shaped by the respondents and not researcher bias, motivation, or interest. This aspect was achieved through the reflexive discussion amongst researchers to ensure that potential biases on meaning interpretation were minimised.

## Findings

### Summary of perceptual, emotional and coping experiences

Thai youth tourists who return from the exchange programmes and from the work and travel programme demonstrated a number of perceptual stages after completing their work, holiday and educational experiences overseas. Their viewpoints were changed within the two broad themes (levels), namely, individual and social levels. Family and school were found to play significant roles and exhibit strong impacts on their perception of their respective home countries. In addition to the changes in perceptual dimension, the returnees experienced various emotions that occurred at different stages. For instance, their emotions were remarkably negative after few weeks to one month upon their return. The feelings mentioned were alienation, loneliness and depression, frustration, anger, boredom with the environment and sadness. Five respondents addressed their willingness to leave the country during this period. During the two to six-month period, returnees felt a mixture of negative feelings that were more dominant than positive ones. Upon entering the seventh and eighth months, the majority of returnees held more positive feelings than negative ones, which indicated improved adaptation at home. They mentioned about having more friends and positive relationships and feeling comfortable with their home environment. Nevertheless, a few negative aspects were still mentioned by the returnees who continued to experience difficulties in terms of fitting in.

With regard to the coping strategies, four types of Adler [19]’s coping strategy were noted by the returnees at different degrees. The data were categorised through thematic coding analysis of the context based from the interviews. In addition, the analysis results were matched with Adler’s coping strategy types, namely, proactive, resocialised, rebellious and alienated. The first two groups, proactive and resocialised, indicate coping with optimism, whilst the latter two, rebellious and alienated, indicate the coping with pessimism. Although many youth tourists faced upon their return, most of them coped with RCS through optimism. The coping strategies of ‘proactive’ and ‘resocialised’ were mostly applied by the Thai youths, with more weight on the resocialising coping mode than proactive coping.

### Graph plotting results

**Fig. 6:**
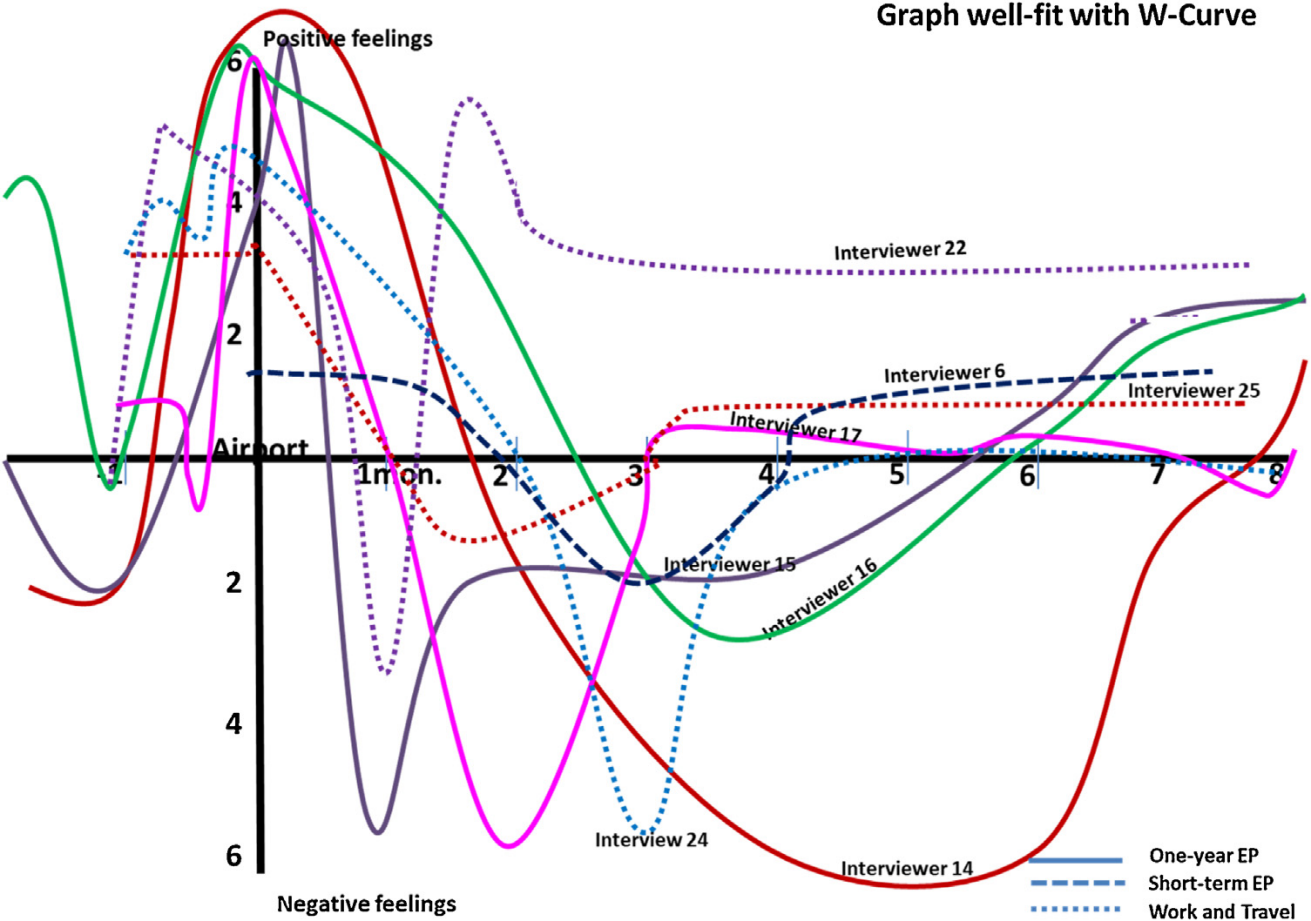
Graphs well-fit with W-curve proposition (2nd U-curve).

Each individual returnee was asked to plot his or her emotional level on to the graph sheet (Fig. 3) where the emotional scale ranged from -6 to +6 (where the minus, zero and positive zones represented negative, neutral and positive feelings, respectively). The setting point of the starting second U-curve is at the time when they landed at their local airport. The plotted graph represented the emotional level of the returnees from the stage at one month of preparations to come home to the stage at eight-month adjustment in the home country compared with the W-curve proposition by Gullahorn and Gullahorn [4]. Fig. 6 illustrates the comparison.

**Fig. 7:**
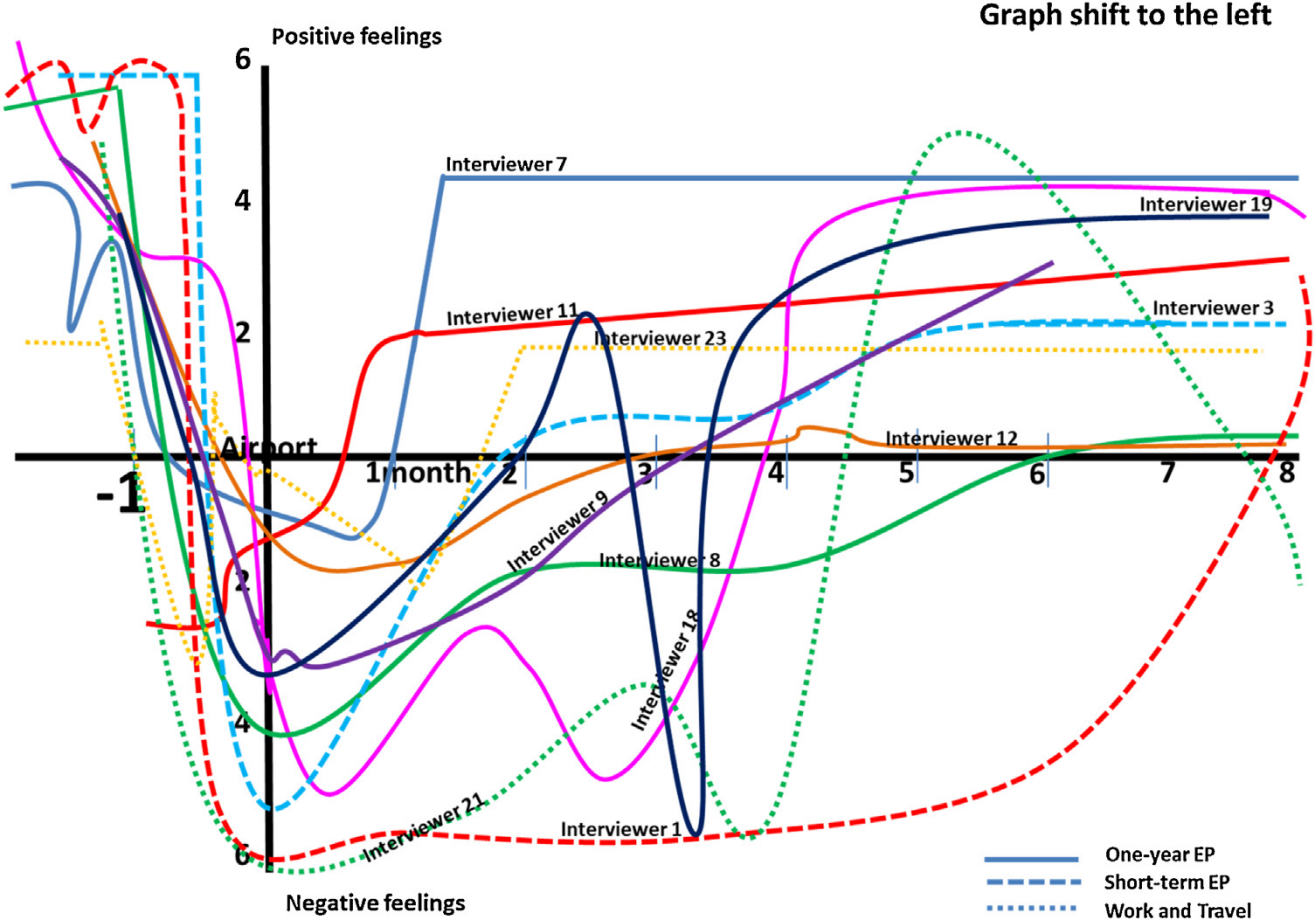
Graphs shift to the left compared with the W-curve proposition.

**Fig. 8:**
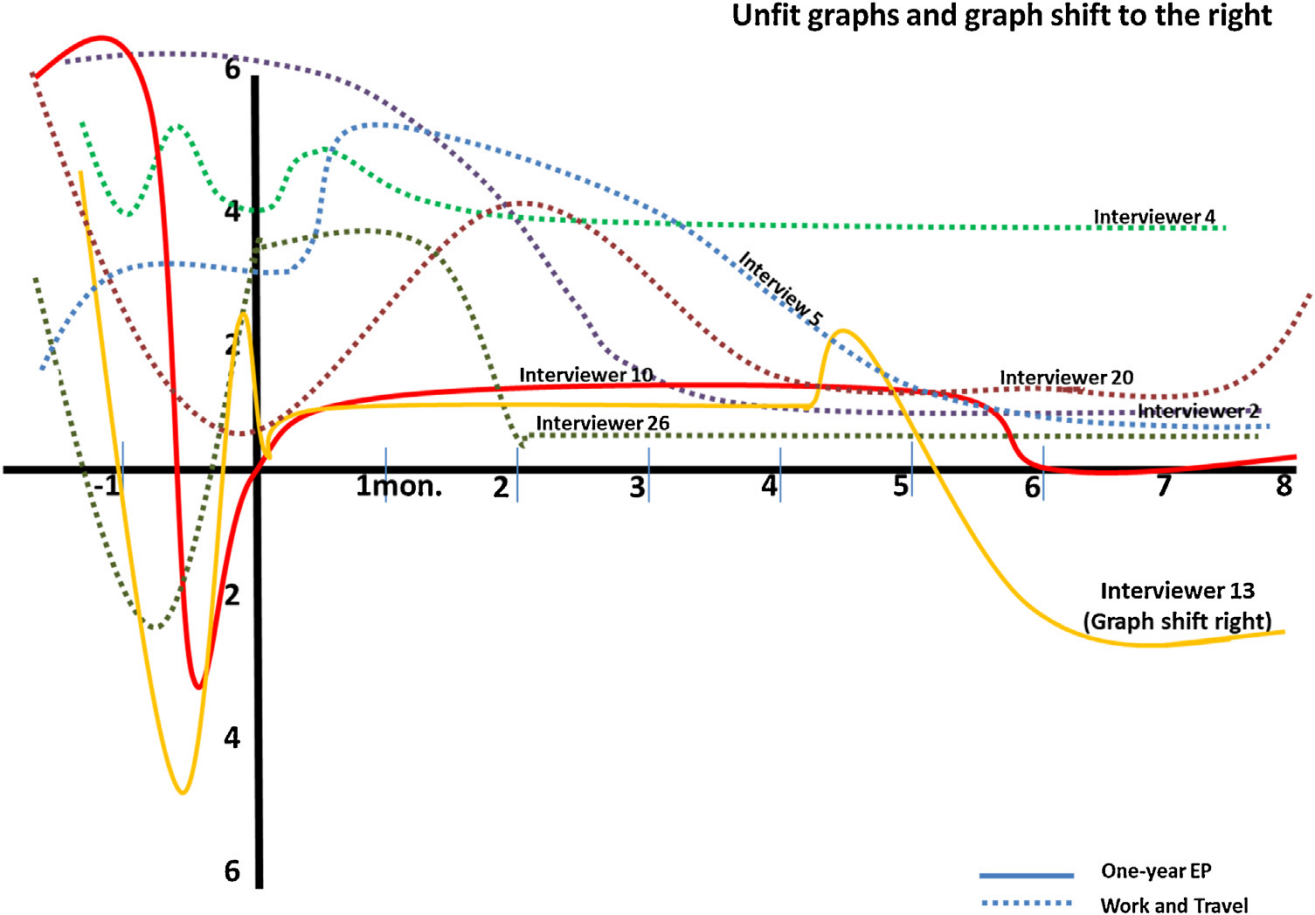
The unfit graphs and one graph shift to the right compared with the W-curve proposition.

Figs. 6, 7 and 8 reveal the three graph types derived from the 26 participants. Firstly, graph fit well with the W-curve proposition (Fig. 6) and presented that the positive emotional stage (honeymoon) lasted for a few weeks upon returning to the home country. The graph significantly dropped to the negative emotional stage for months and slowly shifted back to the positive feeling zone. This type of pattern relatively fits well when compared with the traditional W-curve proposed by Gullahorn and Gullahorn [4] (see Fig. 1).

Secondly, the graph that shifted to the left (Fig. 7) demonstrated the negative emotional stage including the feelings of unwillingness to return home and unhappiness upon return and the first few months in the home country. This left-shifting pattern revealed the lack of honeymoon phase upon return. The curve falling into the negative area reflects their difficulties in their home environment. Moreover, the adjustment stage was varied depending on the degrees and periods plotted by the returnees.

Lastly, the unfit graph and graph that shifted to the right (Fig. 8) are also found. The graph is unfit to the W-curve proposition and shows lack of relationship to RCS. Given the lack of RCS stage, returnees showed their desire to come back to the home environment and appreciated things at home better than before. Nonetheless, the diverse graphs of this pattern can reflect undesired experiences in the host country. Thus, the returnees looked forward to returning home.

### Conclusion and challenges in the RCS study

This paper applies a mixture of qualitative research methods to investigate the RCS experiences of Thai youth tourists. The study reveals that by combining various techniques, rich and comprehensive data can be derived from Thai participants, which yield improved and different patterns from a complex phenomenon, particularly RCS. This paper including the selected methods can contribute to the extended areas of social science and psychology that requires the accuracy of memory recall.

One of the difficulties or limitations found in the studies of culture shock (and potentially RCS) is the use of cross-sectional study rather than longitudinal study [[24], [25], [26]]. In particular, the latter allows changes in the adjustment process that can be improved over time [27] and yields further accurate results. Given that the current research is cross-sectional, the researchers attempt to lessen this limitation by applying sequential procedures. Therefore, multiple methods (i.e. writing an essay, graph drawing activity and conducting a semi-structured interview) are adopted to help the respondents deeply recall and elaborate on their past memories of their overseas experience. The use of multiple methods could help reduce memory bias and distortion and allow for accurate details at each time period. Such multiple methods could also aid in minimising the differential memory effects that possibly occur from the data collected at a particular point in time [24].

Another limitation of this research is related to the composition of the sample. Although the researchers attempted to balance the number of participants who have short-term versus one-year overseas experience, the imbalance of countries visited by the youths remain. In particular, those who went to the US comprise half of the participants, and the results fail to indicate whether people who visited the US portrayed a similar pattern of their experiences amongst themselves. This imbalance may have some impact on the overall result, especially on the ability to distinguish patterns according to the different cultural dimension of visited countries. Therefore, the inclusion of other overseas destinations with different cultural distance could provide additional findings and insight into the RCS study.

Lastly, a general limitation of the RCS studies lies in the mixed use of *psychoemotional* indicators and *behavioural and cognitive* indicators to measure the returnee’s adaptation [27], thereby leading to the vague distinction among the behavioural, cognitive and affective dimensions derived from previous studies. The current study attempts to lessen this limitation by employing the graph drawing procedure to highlight the emotional stage that occurs at each phase of participants’ adaptation and thus supplements the interview method. The interview is also conducted to investigate on the perceptual (cognitive) and behavioural aspect of the returnees’ experiences before and after return. The latter is thematically coded based on the four coping strategies proposed by Adler [19], which comprises four behavioural coping modes of returnees. Thus, these combinations of research methods enable the researchers to separate the two-intertwined indicators in the previous RCS studies and mark a clear distinction between the two aspects.

## Declaration of Competing Interest

The authors declare that they have no known competing financial interests or personal relationships that could have appeared to influence the work reported in this paper.
