# Supplementary material for: Multiple qualitative procedures to elicit reverse culture shock experience
Source: MethodsX. 2019 Dec 16;7:100766. doi: 10.1016/j.mex.2019.12.007 (PMC6992980; doi:10.1016/j.mex.2019.12.007)
Supplement: Supplementary file 1 [file mmc1.doc]

**Appendix 1**

Pre-Activity: Drawing Graph and choosing emotion


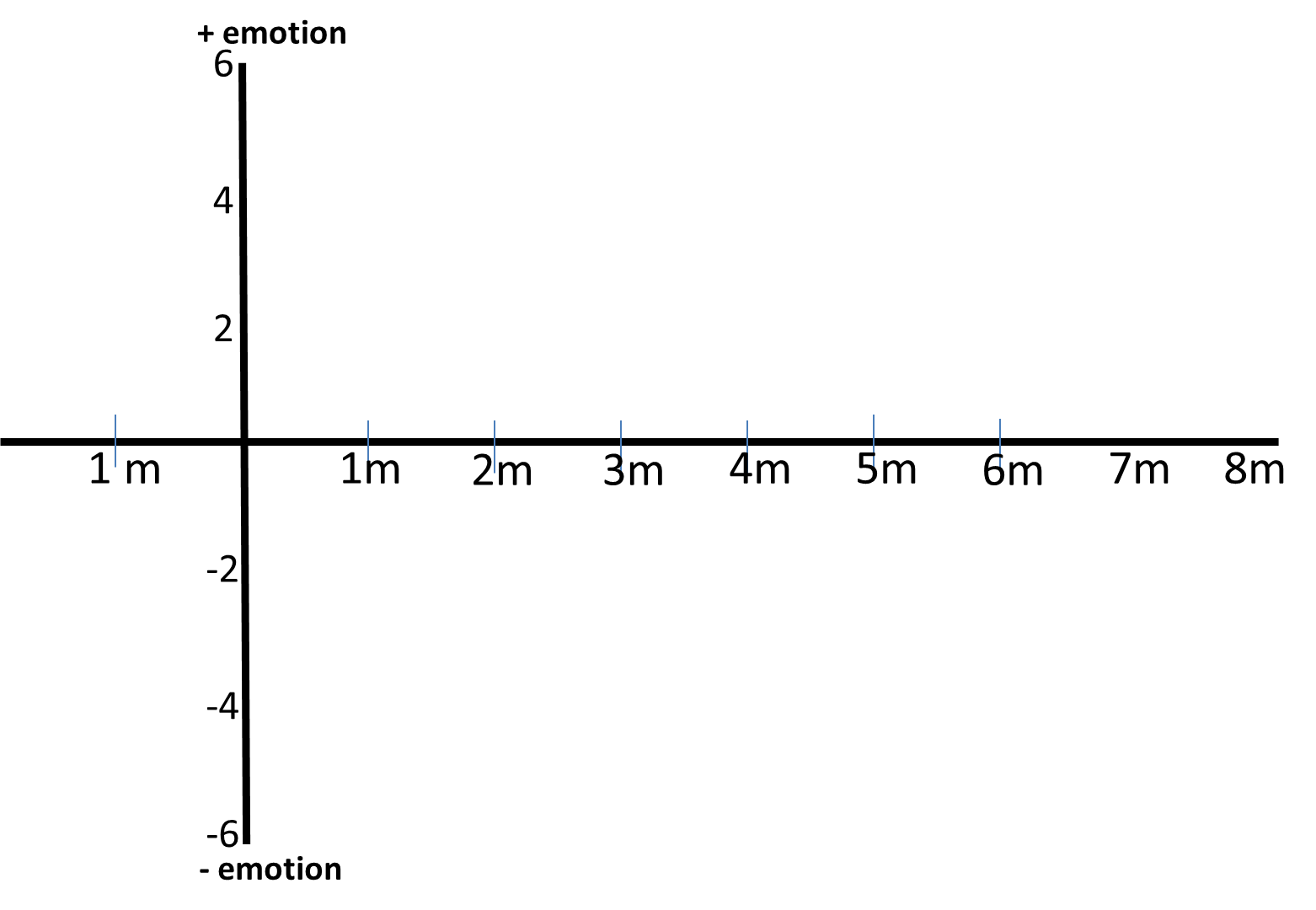


Perceptual and Emotional Set (adapted from Gaw, 2000)


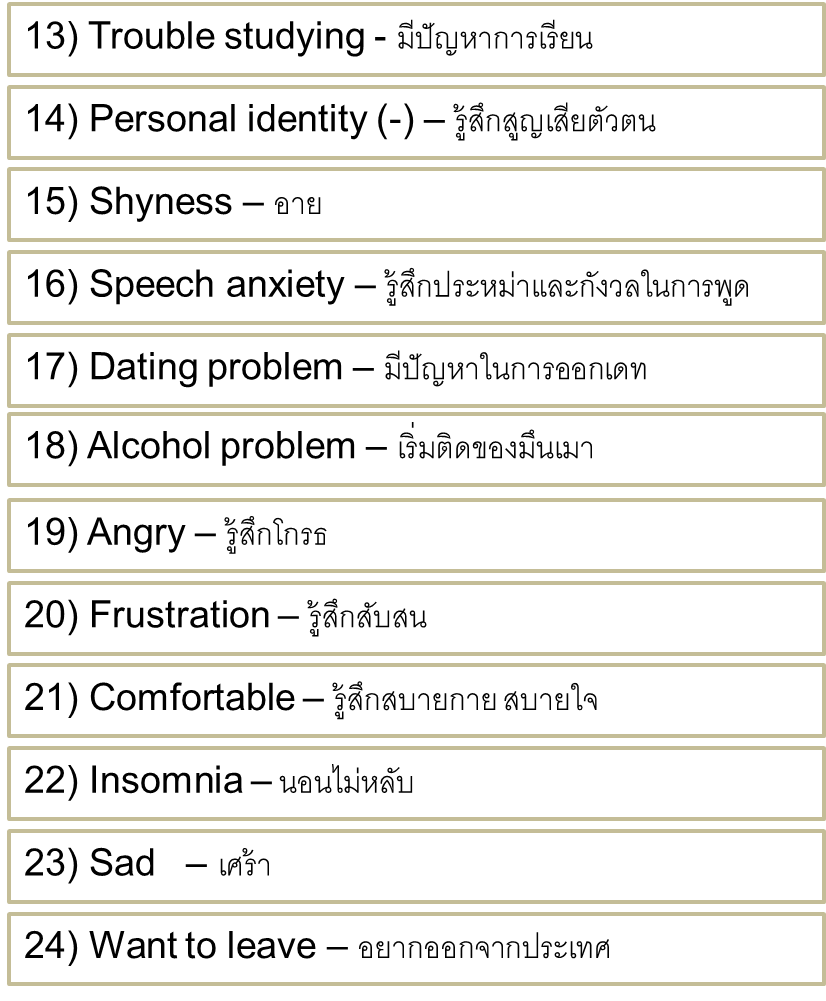

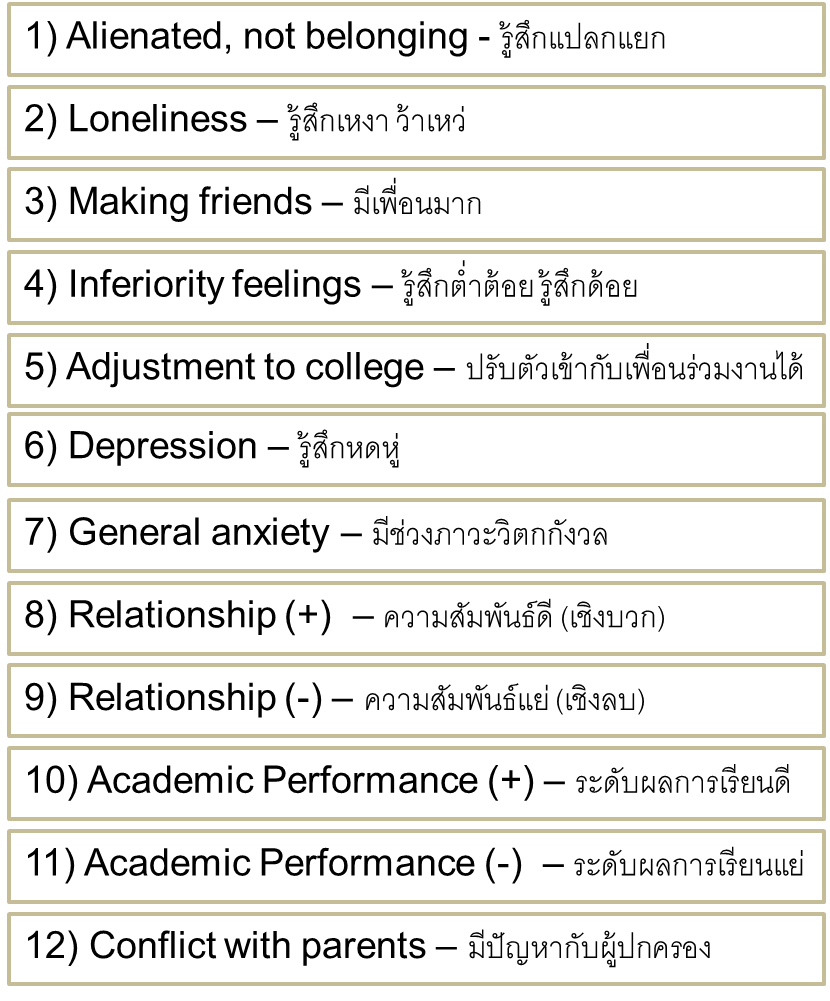


Interview questions (Semi-Structured Interview)

1. What was your expectation that made you went overseas?

คุณคาดหวังอะไรก่อนเดินทางไปต่างประเทศ

1. How did you feel after your return home? (Consulting the drawing graph)

หลังจากที่คุณกลับมาถึงแล้วคุณรู้สึกอย่างไร (สัมภาษณ์ร่วมกับกราฟที่วาดไว้ก่อนหน้านี้)

- 1. Probing: Can you explain each stage of your drawing and why you label the particular stage with those emotional / perceptual terms?

คำถามลงลึก กรุณาอธิบายรายละเอียดว่าเหตุใดในแต่ละช่วงเวลาคุณจึงเลือกใช้คำแสดงอารมณ์ หรือคำแสดงสภาวะการณ์แบบนี้

- 1. Are the feelings in that stage on the graph applied to what you feel about your […]?

[…] indicates the following dimensions:

ความรู้สึกในแต่ละช่วงเวลาบนรูปกราฟ เกิดขึ้นกับด้านต่างๆ เหล่านี้ด้วยใช่หรือไม่

- Family ครอบครัว
- Relationship ความสัมพันธ์
- Work การงาน
- Daily Lifestyle การใช้ชีวิตประจำวัน
  1. How did you cope with each stage after return?

หลังจากเดินทางกลับมาแล้ว คุณมีวิธีจัดการตัวเองในแต่ละช่วงเวลาอย่างไร

1. Now since you have come back, is it worth going overseas, both financially and non-financially (e.g. relationship, mental, experience etc.)?

หลังจากเดินทางกลับมาแล้ว คุณคิดว่าการเดินทางครั้งนี้คุ้มค่าหรือไม่เมื่อเทียบกับสิ่งที่เสียไปและสิ่งได้รับมา ทั้งด้านการเงินและด้านอื่นๆ (เช่น ความสัมพันธ์ สุขภาพจิต ประสบการณ์)

1. Would you have still chosen to go, knowing that you would experience the unpleasant feelings after coming back?

หากคุณทราบว่าคุณต้องประสบกับความรู้สึกในเชิงลบหลังจากกลับมาแล้ว ถ้าย้อนเวลากลับไปได้ คุณยังเลือกที่จะเดินทางไปต่างประเทศอยู่หรือไม่

Thank you for your collaboration in this study.

ขอขอบคุณสำหรับความอนุเคราะห์ที่เข้าร่วมให้ข้อมูลการวิจัยครั้งนี้
